# A tissue-silicone integrated simulator for right ventricular pulsatile circulation with severe functional tricuspid regurgitation

**DOI:** 10.1038/s41598-024-55058-w

**Published:** 2024-03-01

**Authors:** Jumpei Takada, Hayato Morimura, Kohei Hamada, Yusei Okamoto, Shiho Mineta, Yusuke Tsuboko, Kaoru Hattori, Kiyotaka Iwasaki

**Affiliations:** 1 Department of Modern Mechanical Engineering, School of Creative Science and Engineering, Waseda University, Tokyo, Japan; 2 Department of Integrative Bioscience and Biomedical Engineering, Graduate School of Advanced Science and Engineering, Waseda University, Tokyo, Japan; 3 Cooperative Major in Advanced Biomedical Sciences, Joint Graduate School of Tokyo Women’s Medical University and Waseda University, Waseda University, Tokyo, Japan; 4 Waseda Research Institute for Science and Engineering, Waseda University, Tokyo, Japan; 5 Department of Modern Mechanical Engineering, Graduate School of Creative Science and Engineering, Waseda University, Tokyo, Japan; 6 Institute for Medical Regulatory Science, Waseda University, Tokyo, Japan

**Keywords:** Valvular disease, Experimental models of disease, Biomedical engineering

## Abstract

There is a great demand for development of a functional tricuspid regurgitation (FTR) model for accelerating development and preclinical study of tricuspid interventional repair devices. This study aimed to develop a severe FTR model by creating a tissue-silicone integrated right ventricular pulsatile circulatory simulator. The simulator incorporates the porcine tricuspid annulus, valve leaflets, chordae tendineae, papillary muscles, and right ventricular wall as one continuous piece of tissue, thereby preserving essential anatomical relationships of the tricuspid valve (TV) complex. We dilated the TV annulus with collagenolytic enzymes under applying stepwise dilation, and successfully achieved a severe FTR model with a regurgitant volume of 45 ± 9 mL/beat and a flow jet area of 15.8 ± 2.3 cm^2^ (n = 6). Compared to a normal model, the severe FTR model exhibited a larger annular circumference (133.1 ± 8.2 mm vs. 115.7 ± 5.5 mm; *p* = 0.009) and lower coaptation height (6.6 ± 1.0 mm vs. 17.7 ± 1.3 mm; *p* = 0.003). Following the De-Vega annular augmentation procedure to the severe FTR model, a significant reduction in regurgitant volume and flow jet area were observed. This severe FTR model may open new avenues for the development and evaluation of transcatheter TV devices.

## Introduction

The tricuspid valve (TV) possesses a unique morphology that includes three leaflets, the TV annulus, the chordae tendineae, which connect the leaflets to papillary muscles on the right ventricular free wall^1–3^. In the United States, more than 1.6 million patients suffer from moderate or severe tricuspid regurgitation (TR)^3^. Functional tricuspid regurgitation (FTR) accounts for 85–90% of all severe TR cases^3^, typically induced by annular dilation and subsequent leaflet tethering. Clinically, interventions for the TV are often under-conducted and initiated too late^4^. Current guidelines for managing valvular disease recommend early surgical intervention for patients with symptomatic severe TR to prevent irreversible right ventricle dysfunction and organ damage^5,6^. Common surgical treatments for FTR include annuloplasty, which uses an artificial ring to correct the size and shape of the TV annulus^7,8^. With significant advancements in transcatheter aortic valve replacement and clinical trials of emerging transcatheter mitral valve repair devices^9,10^, research into transcatheter tricuspid interventions has gained considerable attention, especially for patients who are inoperable and have severe TR^11,12^. For evaluating efficacy and safety, testing with a clinically relevant FTR model is necessary. However, current in vitro and ex vivo models fall short in replicating severe TR with a regurgitant volume of more than 45 mL/beat^13,14^. Due to the extreme challenge of developing severe FTR in animal models, there is a pressing need to create an experimental system that can morphologically and physiologically simulate severe FTR, which is vital for evaluating transcatheter TV repair devices.

In this study, we aimed to develop a severe FTR model by creating a pulsatile circulatory simulator that incorporates a novel tissue-silicone integrated right ventricular model using a porcine TV, annulus, and ventricular wall. We demonstrate the feasibility of this tissue-silicone integrated simulator for right ventricular pulsatile circulation with severe FTR by assessing changes in hydrodynamic performances and morphology following an annulus augmentation procedure.

## Methods

Given that the porcine heart valves obtained from slaughterhouses do not exhibit any pathological conditions, we devised a method to create a model of a dilated valve annulus using a porcine heart. This was achieved by partially degenerating the collagen tissue of the porcine TV annulus using an enzymatic solution, followed by enlarging the annulus using an annulus dilator. Our goal was to demonstrate the feasibility of creating a model for severe FTR. This process involved integrating the dilated annulus of RV tissue into a dilated silicone model of the right ventricle. The feasibility of the simulator was demonstrated by assessing the changes in hydrodynamic performances and morphology following an annular augmentation with the De-Vega procedure.

### Development of a dilated annulus tissue model

**Figure 1:**
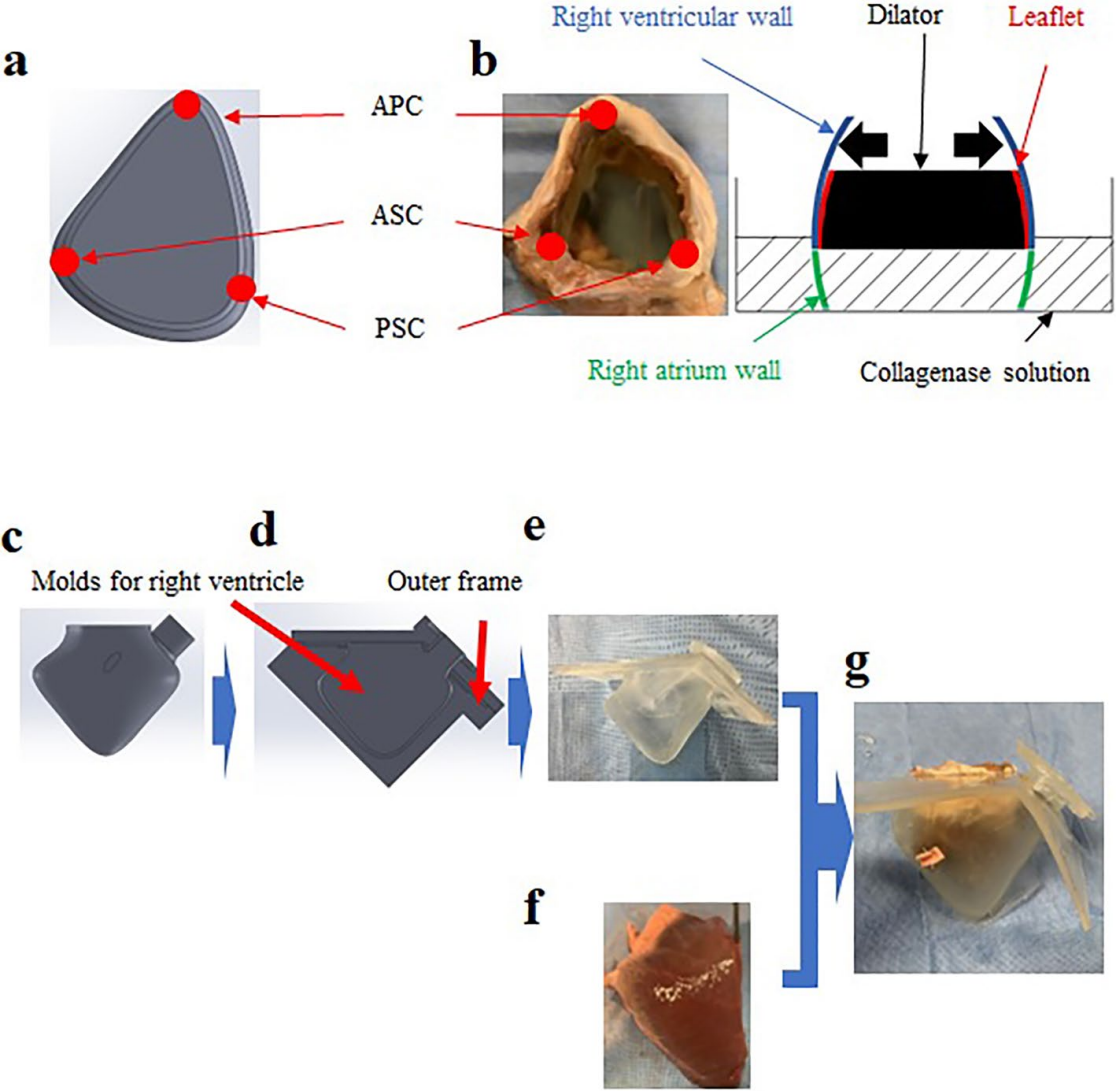
A method for developing tissue-silicone integrated right ventricular model. (**a**) A dilator mimicking the human annulus morphology of FTR, (**b**) Method to insert a dilator into the tricuspid valve annulus. (**c**) Computer-aided design of a right ventricular model, (**d**) The gap between the right ventricular model and the outer frame, (**e**) A silicone right ventricular model, (**f**) The right ventricle tissue, (**g**) A tissue-silicone integrated model. FTR: Functional tricuspid regurgitation, APC: Anterior–posterior commissure, ASC: Anterior-septal commissure, PSC: Posterior-septal commissure.

Twelve porcine hearts (aged 35–38 weeks old, weighing 90–99 kg) without pathological conditions were obtained from a local abattoir. Hearts lacking papillary muscles or those with more than four valve leaflets were excluded. Initially, the TV annulus with the right ventricular wall was trimmed from the porcine heart. The TV annulus was excised from the right atrial wall, leaving a 10-mm tissue margin from the annulus for suturing to a silicone RV model. Right ventriculotomy was limited to the RV outflow-free wall to preserve RV anatomy and ensure physiological leaflet dynamics. To develop the dilated annulus in RV tissue, the TV annulus was treated with collagenolytic enzymes (C6885-500MG, Sigma-Aldrich., St. Louis, USA) to induce degeneration of the annulus tissue. The annulus was then incrementally dilated using a series of dilators. Considering that the TV annulus in FTR is likely to dilate asymmetrically towards the commissure between the anterior and posterior leaflets^15^, we designed a specific dilator as shown in Fig. 1a. The size of the dilator was selected based on the initial measurement of the distance between the anteroseptal and anteroposterior commissures^16^. As the circumference of the annulus in FTR typically increases by approximately 16%^17^, we chose a dilator with a circumference 10% larger than the measured TV annulus as the first step. The annulus, once dilated, was immersed in the collagenase solution for 10 min (Fig. 1b). Subsequently, the dilation processes were continued using a second dilator with circumference 20% larger than the measured TV annulus. Finally, to achieve a circumference approximately 16% larger than the original, a third dilator with a 30% increase in circumference relative to the measured TV annulus was used.

### Construction of a tissue-silicone integrated right ventricular model

**Figure 2:**
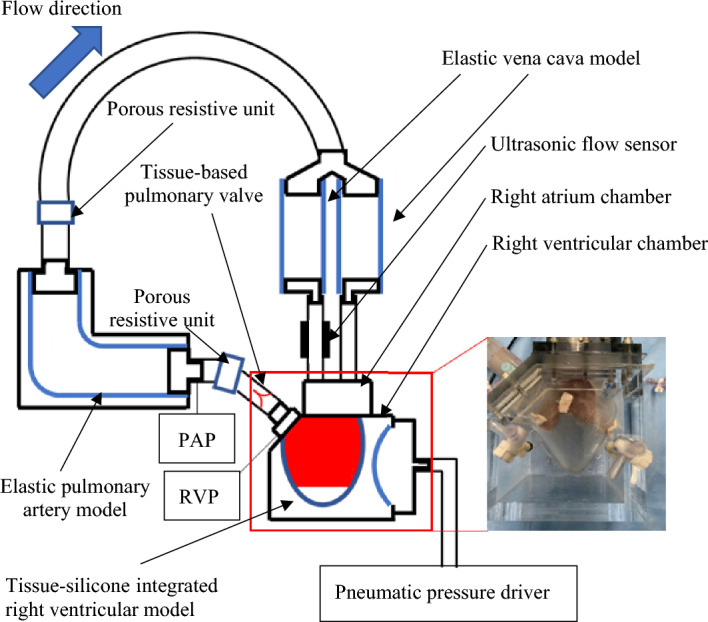
A tissue-silicone integrated pulsatile right ventricular circulatory system incorporating a tricuspid valve. PAP; Pulmonary artery pressure, RVP; Right ventricular pressure.

Firstly, we designed a right ventricular model using a computer-aided design (CAD) software (Solidworks 2019, Dassault Systemes Solidworks Corporation, Paris, France) (Fig. 1c). The CAD data were then exported to a 3D printer (Eden260vs, Stratasys, Valencia, CA, USA). Secondly, a rigid right ventricular model was constructed using a stereolithography resin (FullCure720, Altech, Tokyo, Japan). Thirdly, a wax model of identical shape was constructed using a casting technique, and an outer frame model, enlarged by 3 mm outward, was fabricated using stereolithography. Fourthly, using the wax mold and the outer frame, an elastic right ventricular model was fabricated with silicone (Shin-Etsu Silicone [KE-1603-A: KE-1603-B: KF-96-50CS; 10:10:8], Shin-Etsu Chemical, Tokyo, Japan) (Fig. 1d and e). In this study, excised right ventricular tissue was sutured onto the inner surface of the elastic right ventricular model. To induce uniform contraction of the right ventricular model from the outside using pneumatic pressure, the thicknesses of both the excised septum and the free wall were adjusted to be equivalent. From a manufacturing ease standpoint, a thickness of 3 mm was deemed appropriate. Additionally, the right ventricular model with this 3-mm thickness did not tear when the annulus of the porcine tricuspid valve was sutured onto the silicone model. An elastic right ventricle model was fabricated based on the referenced geometry of 40 healthy adults^18,19^. For the dilatated silicone RV model, the anteroposterior commissure of the TV annulus was dilated to morphologically replicate the enlargement of the right ventricle in patients with severe FTR. The annulus perimeter and internal volume of the dilated silicone RV model were set to values 17% and 40% larger than those of the normal model, respectively, based on values from the literatures^17,20^. The RV tissues were sutured to the silicone RV models using 4–0 proline suture (Fig. 1f), culminating in the construction of the tissue-silicone integrated RV model (Fig. 1g), which was installed in an airtight acrylic chamber (Fig. 2). The three-dimensional shape of both the normal and dilated tissue-silicone integrated RV models, including RV volume, the straight-line distances between papillary muscles, and the distance from the annular plane to each papillary muscle, were measured using micro-CT (TDM 1300-IS, Yamato Scientific Co., Ltd., Japan) (Table 1). The RV volume in the FTR model was larger than that in the normal model (Table 1). The lengths of the anterior-septal and posterior-septal papillary muscles in the FTR model were greater than those in the normal model (Table 1). Although there was no difference in the distance from the annular plane to each papillary muscle between the FTR and normal models (Table 1), the distance between the anterior and posterior papillary muscles was significantly larger in the FTR model.

**Table 1:** Right ventricular volumes and morphologies of the tricuspid valve complex in normal and functional tricuspid regurgitation models.

	Measurement value		*p* value
	Normal	FTR	Normal vs FTR
RV volume mL	110.2 ± 15.3	170.3 ± 11.4	< 0.01
APM-PPM distance mm	18.2 ± 4.1	15.3 ± 3.8	0.05
APM-SPM distance mm	25.6 ± 3.3	26.3 ± 2.8	0.1
PPM-SPM distance mm	17.3 ± 3.9	22.8 ± 1.5	< 0.01
Annulus-APM distance mm	16.3 ± 1.3	15.7 ± 1.4	0.2
Annulus -PPM distance mm	14.8 ± 1.1	14.6 ± 0.7	0.3
Annulus -SPM distance mm	14.4 ± 2.1	14.6 ± 0.9	0.3

*FTR* functional tricuspid regurgitation, *RV* right ventricle, *APM* anterior papillary muscle, *PPM* posterior papillary muscle, *SPM* septal papillary muscle.

### Development of a tissue-silicone integrated right ventricular pulsatile circulatory system

The pulsatile circulatory system we constructed comprised a porcine TV, the tissue-silicone integrated RV model situated within the right ventricular chamber, a pulmonary valve of 33-mm diameter made of bovine pericardium using our previously reported method^21^ (corresponding to the human right ventricular outflow tract diameter^22^), a porous resistive unit, an elastic pulmonary artery (PA) model, an elastic vena cava model, a right atrium chamber, and a pneumatic console (VCT-50 (χ), Nipro, Osaka, Japan) used to drive the right ventricular model (Fig. 2). The PA pressure was regulated using multiple porous resistors with varying numbers of pores, in addition to adjusting the elastic modulus of the elastic PA model. A 0.9% physiological saline solution was used as the working fluid. Flow through the TV was measured using an ultrasonic flow probe (ME-PXN ME19PXN325; Transonic, NY, USA). We confirmed that the same flow rate passed through the two channels, as their diameters and lengths were maintained identically. Consequently, we measured the flow rate in one of these channels and then doubled this value to represent the total flow rate for the experiment. Pressure was measured using pressure transducers (TruWave, Edwards, California, USA). Hemodynamic data collected using measurement software (LabView; National Instruments, Austin, USA) was subsequently calculated by averaging the forward flow (valve inflow) and backflow (valve regurgitation) data from six consecutive waveforms using numerical analysis software (MATLAB, The MathWorks, Natick, USA).

### Test procedures

**Figure 3:**
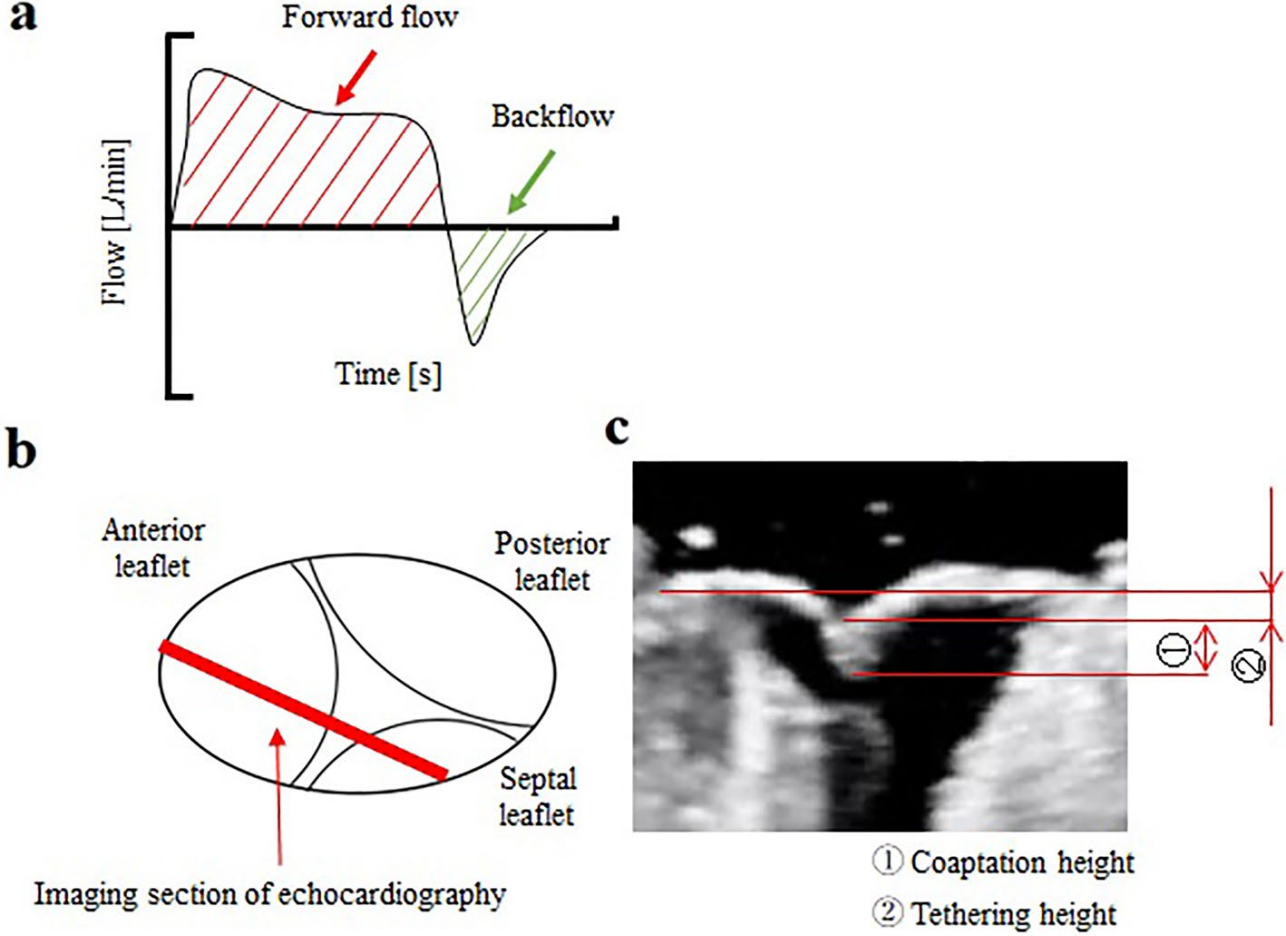
Measurement of flow parameters and morphologies of the tricuspid valves. (**a**) Forward flow and backflow of a tricuspid valve, (**b**) Imaging section of echocardiography (**c**) Coaptation height and tethering height.

First, the hydrodynamics of six normal tricuspid valve models including backflow, the percentage of backflow to forward flow, and RV and PA pressure were measured under a forward flow rate of 5.0L/min at a pulse rate and systolic fraction of 70 bpm and 35%, respectively. The target systolic/diastolic right ventricular and pulmonary artery pressures were set at 20–30/0–5 mmHg and 15–30/2–8 mmHg, respectively (Fig. 3a)^23,24^. The percentage of backflow to forward flow was defined using the following equation.

1 $$\begin{array}{c}Percentage\, of\, backflow\, to\, forward\, flow\,=\,\frac{Backflow \left[mL\right]}{Forward\, flow \left[mL\right]}\times 100\end{array}$$

Regurgitation is classified as mild when backflow is less than 30 mL/beat, moderate for volumes between 30 and 44 mL/beat, and severe when 45 mL/beat or more^5,6^. In this study, under the forward flow rate of 5.0/min and the pulse rate of 70 bpm, regurgitation is classified as mild when the percentage of backflow to forward flow is below 42%, moderate between 43 and 62%, and severe when 63% or more. Jet flow area at the time of maximal systolic closure was measured using color Doppler mode on a cardiology ultrasound system (EPIQ CVx 3D, Philips, Amsterdam, Netherlands) with a transthoracic probe (X5-1). The transthoracic probe was positioned on the right atrium chamber. The frame rate was set to 15 Hz with an imaging depth of 17 cm, and the maximum flow velocity was defined as 61.6 cm/s. Severe regurgitation was defined as a jet flow area exceeding 10 cm^2^^25^. Coaptation height of the leaflets and the tethering height from the annular plane to the coaptation of the leaflets at the time of maximal systolic closure were measured using B-mode on the cardiology ultrasound system (Fig. 3b,c). The transthoracic probe was positioned perpendicular to the valve onto the right atrium chamber. The distances of the papillary muscles were measured on a coronal plane of the RV during peak regurgitation, where the longest coaptation of septal and anterior leaflets occurred. All echocardiographic measurements were performed by the same examiners to ensure reproducibility. Two cardiac surgeons (the authors, K.H. and H.M.), experienced in echocardiographic examination, and a medical engineering student (the author, J.T.) analyzed the data. A high-speed camera (VW9000, KEYENCE, Osaka, Japan) was set above the tricuspid valve model to capture the behavior of the TV leaflet during heartbeats from the right atrial side. The circumferences of the TV annulus were measured using imaging software (VW9000-Motion analyzer, KEYENCE, Osaka, Japan) from the right atrial side. Next, we tested six FTR models. Clinically, FTR is often associated with increased pulmonary arterial pressure. Therefore, in our FTR models, pulmonary arterial pressure was elevated by increasing flow resistance. After collecting data from these FTR models, the De-Vega annular augmentation procedure^26^ was applied. This procedure involved using 4–0 proline sutures on the annulus, overlaying anterior and posterior leaflets of the FTR models.

### Statistical analysis

Continuous data were presented as means ± standard deviations. Statistical analyses were performed using a software (SPSS 26, IBM, Tokyo, Japan). Normality and equality of variances were evaluated using the Shapiro–Wilk and Levene tests, respectively^27,28^. The measured data were compared among the normal model, the TR model, and the TV annulus augmentation model using the One-Way ANOVA method and Bonferroni correction^29,30^. Bonferroni’s correction factor is 3. A *p-value* lower than 0.05 was considered statistically significant.

## Results

### Hydrodynamic measurements of the normal model

**Figure 4:**
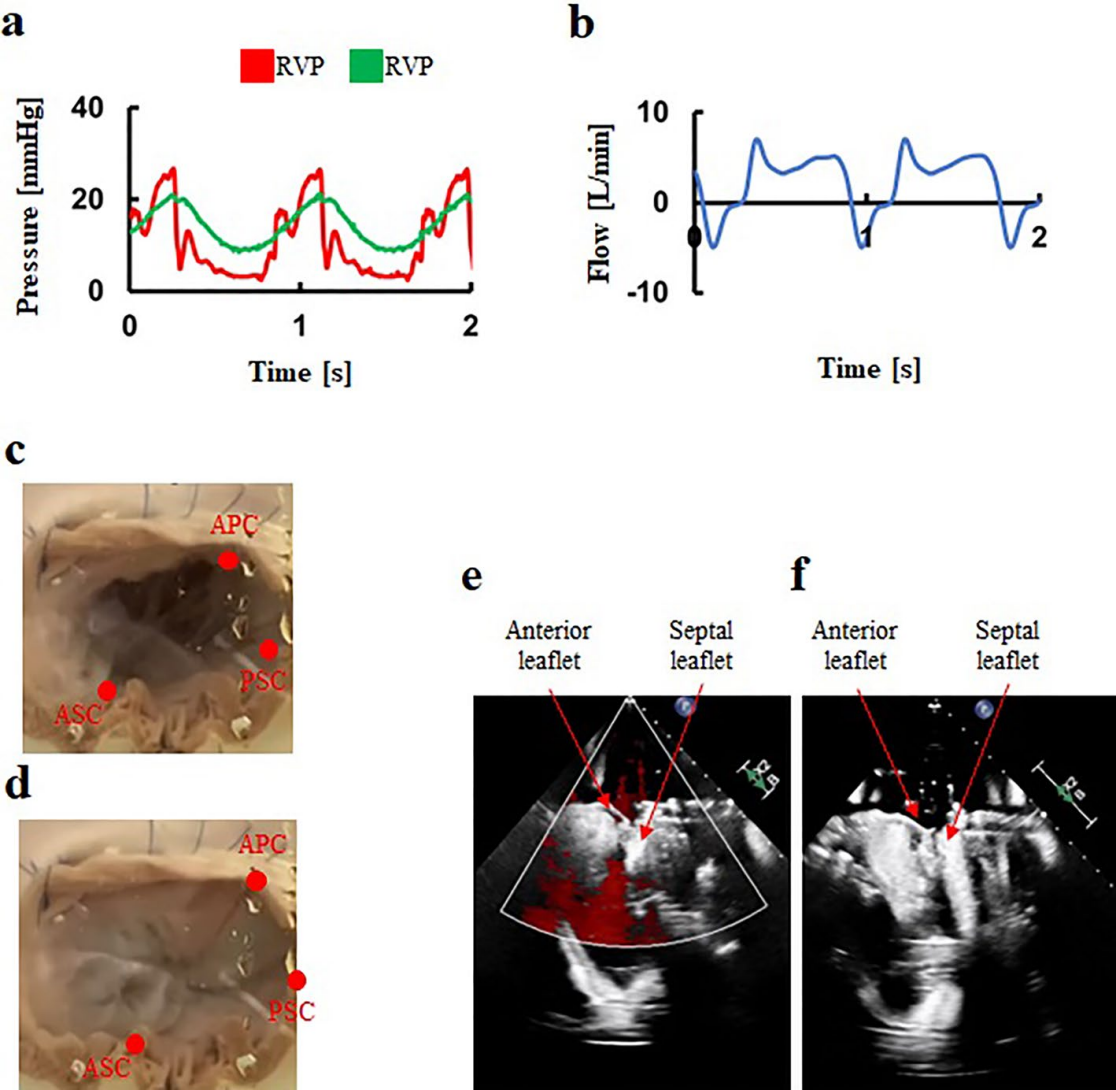
A representative hemodynamics and behaviors in the normal model. (**a**) Pressure waveforms, (**b**) Flow waveform, (**c**) Tricuspid valve at diastole, (**d**) Tricuspid valve at systole, (**e**) Transvalvular jet flow at systole, (**f**) Leaflet tethering at systole. RVP: Right ventricular pressure, PAV: Pulmonary artery pressure, APC: Anterior–posterior commissure, ASC: Anterior-septal commissure, PSC: Posterior-septal commissure.

The tissue-silicone integrated simulator generated physiological RV and PA pressures under the mean forward flow of 5.0L/min (Fig. 4a). The backflow and percentage of backflow to forward flow were 8.6 ± 2.9 mL/beat and 13.2 ± 1.5% (Fig. 4b, Table 2). The dynamics of the TV leaflets were captured using the high speed camera (Fig. 4c and d).

**Table 2:** Hemodynamics and morphologies of the normal tricuspid valve model, the functional tricuspid regurgitation model, and the annular augmentation model using the De-Vega procedure.

	Measurement value			*p* value		
	Normal	FTR	De-Vega	Normal vs FTR	FTR vs De-Vega	De-Vega vs Normal
Forward flow mL/beat	70 ± 6	70 ± 13	66 ± 7	0.5	0.2	0.1
Backflow mL/beat	9 ± 3	45 ± 9	17 ± 4	< 0.01	< 0.01	< 0.01
RVP mmHg (Systole/ diastole)	26.3 ± 1.2/ 3.4 ± 0.8 [Mean]11.0	35.8 ± 2.1/ 11.2 ± 1.0 [Mean]19.4	31.8 ± 1.8/ 8.8 ± 0.2 [Mean]16.4	< 0.01	< 0.01	< 0.01
PAP mmHg (Systole/ diastole)	19.7 ± 1.1/ 9.3 ± 0.4 [Mean]12.8	27.7 ± 1.2/ 20.5 ± 0.3 [Mean]22.9	20.9 ± 0.4/ 12.7 ± 0.8 [Mean]15.4	< 0.01	< 0.01	< 0.01
Percentage of backflow to forward flow %	13.2 ± 1.5	65.4 ± 7.4	25.1 ± 5.5	< 0.01	< 0.01	< 0.01
Jet flow area cm^2^	3.5 ± 1.2	15.8 ± 2.3	8.8 ± 3.5	< 0.01	< 0.01	< 0.01
Annular circumference mm	115.7 ± 5.5	133.1 ± 8.2	111.5 ± 8.3	< 0.01	< 0.01	0.07
Coaptation height mm	17.7 ± 1.3	6.6 ± 1.0	12.8 ± 1.9	< 0.01	< 0.01	< 0.01
Tethering height mm	2.2 ± 1.7	7.2 ± 1.5	5.2 ± 2.1	< 0.01	0.04	< 0.01

*FTR* functional tricuspid regurgitation, *RVP* right ventricle pressure, *PAP* pulmonary artery pressure.

Echocardiography revealed adequate coaptation of leaflets at TV closure (Fig. 4e, and f, Supplementary figures 1-3). Parameters including backflow, the ratio of regurgitant to forward flow rate, jet flow area, annular circumference, coaptation height, tethering height, RV volume, straight-line distances between papillary muscles, and the distance from the annular plane to each papillary muscle (Tables 1 and 2) were all within the normal ranges observed in clinical situations.

### Hemodynamic measurements of the FTR model

**Figure 5:**
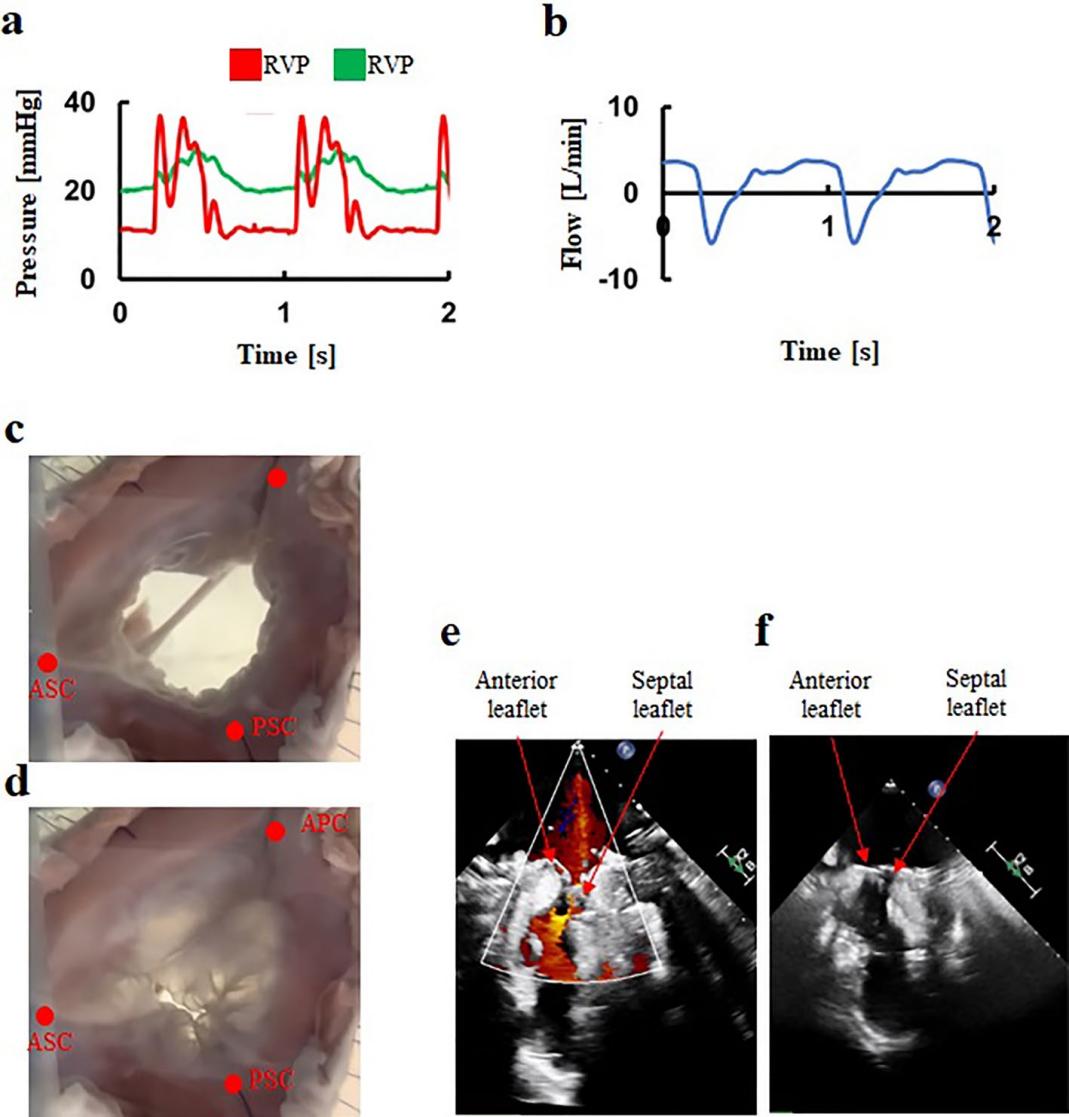
An extreme hemodynamics and behaviors in the FTR model. (**a**) Pressure waveforms, (**b**) Flow waveform, (**c**) Tricuspid valve at diastole, (**d**) Tricuspid valve at systole, (**e**) Transvalvular jet flow at systole, (**f**) Leaflet tethering at systole. FTR: Functional tricuspid regurgitation, RVP: Right ventricular pressure, PAV: Pulmonary artery pressure, APC: Anterior–posterior commissure, ASC: Anterior-septal commissure, PSC: Posterior-septal commissure.

By increasing peripheral resistance, RV and PA pressures in the FTR model increased compared to those in the normal model (Fig. 5a). The tissue-silicone integrated simulator incorporating the FTR model successfully produced severe FTR, with a regurgitant volume of 45±9 mL/beat and a percentage of backflow to forward flow of 65.4 ± 7.4 % (Fig. 5b, Table 2). The dynamics of the TV leaflets were shown in Fig. 5c and d. Echocardiography demonstrated coaptation failure and leaflet tethering at valve closure in the FTR model (Fig. 5e, and f,﻿ Supplementary figures 1-3). The lack of coaptation was exemplified in Fig. 5f. The jet flow area, measuring 15.8 ± 2.3 cm^2^ was indicative of severe FTR and larger in the FTR model than in the normal model (Table 2). The annular circumference was also larger in the FTR model compared to the normal model (Table 2). The coaptation height in the FTR model was lower, and the tethering height was greater than those in the normal model (Table 2).

### Hemodynamic measurement of the annular augmentation model

**Figure 6:**
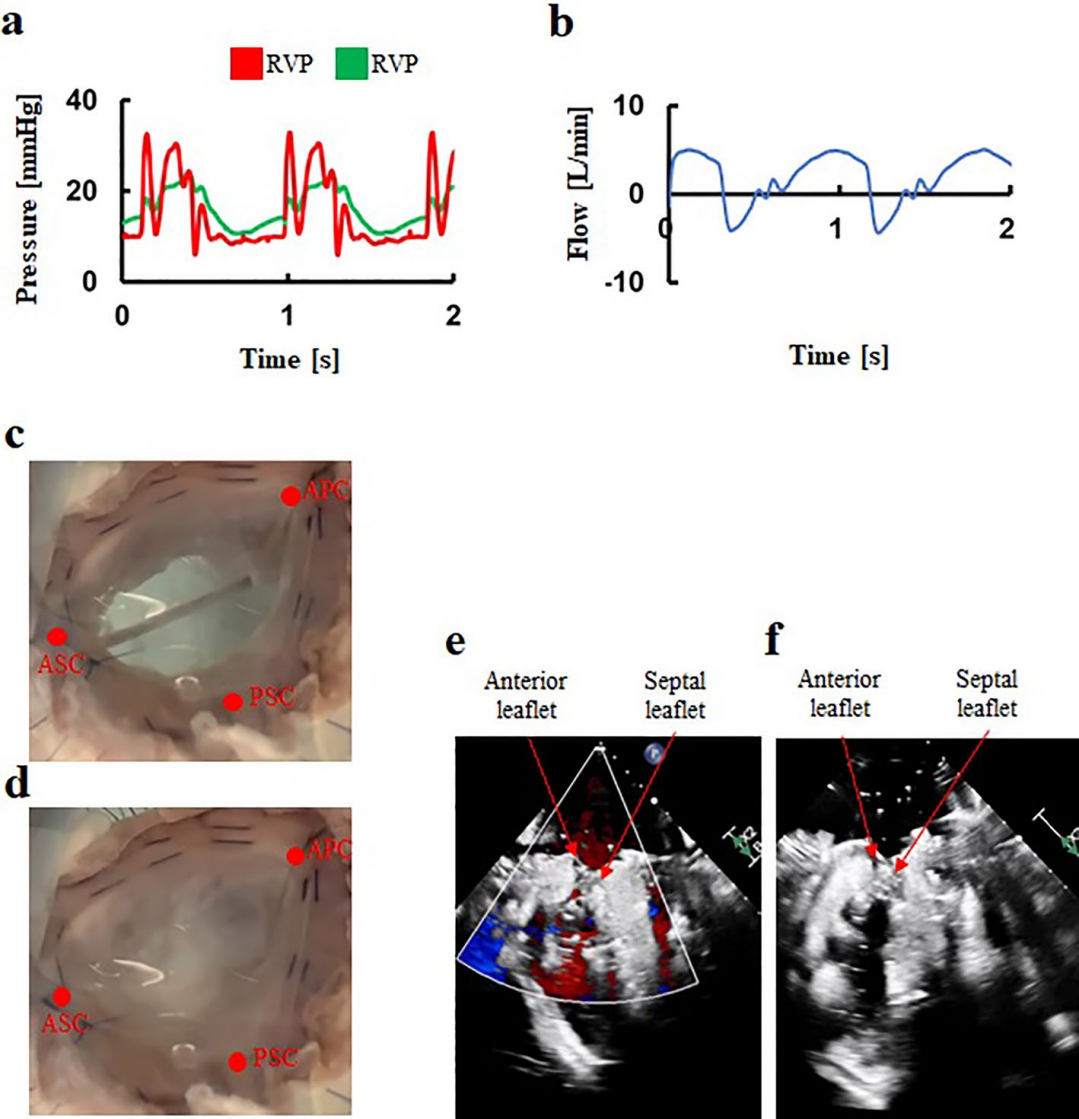
A representative hemodynamics and behaviors in the annular augmentation model using the De-Vega procedure. (**a**) Pressure waveforms, (**b**) Flow waveform, (**c**) Tricuspid valve at diastole, (**d**) Tricuspid valve at systole, (**e**) Transvalvular jet flow at systole, (**f**) Leaflet tethering at systole. RVP: Right ventricular pressure, PAV: Pulmonary artery pressure, APC: Anterior–posterior commissure, ASC: Anterior-septal commissure, PSC: Posterior-septal commissure.

The flow and pressure waveforms of the tissue-silicone integrated simulator, after applying the De-Vega procedure to the FTR models, indicated decreases in backflow, percentage of backflow to forward flow, and jet flow area (Fig. 6a and b). The backflow and the percentage of backflow to forward flow rate were 17.1 ± 4.3 mL/beat and 25.1 ± 5.5%, respectively, corresponding to mild FTR. However, these values in the annular augmentation model using the De-Vega procedure were higher than those in the normal model (Table 2). The dynamics of the TV leaflets were shown in Fig. 6c and d. Although the De-Vega procedure increased the coaptation height compared to the FTR model, it did not alleviate leaflet tethering, as the volume of the dilated RV model could not decrease after the De-Vega procedure in our system (Fig. 6e, and f, ﻿Supplementary figures 1-3). The annular circumference in the annular augmentation model using the De-Vega procedure was smaller than in the FTR model (Table 2). There was no significant difference in the annulus circumference between the annular augmentation model using the De-Vega procedure and the normal model (Table 2).

## Discussion

We have successfully developed a severe FTR model by creating a pulsatile circulatory simulator that incorporates a novel tissue-silicone integrated right ventricular model. This model utilized a porcine dilated TV annulus and ventricular wall as one continuous piece of tissue. We devised a method to create a model of a dilated valve annulus by partially degenerating the annulus collagen using an enzymatic solution, followed by enlarging the annulus with a specially designed annulus dilator. Integrating the dilated annulus of RV tissue into a dilated silicone model of the right ventricle enabled the production of an FTR model. The feasibility of FTR model was demonstrated by data showing that the FTR model produced a severe grade of regurgitant volume of 45 ml/beat and a flow jet area of more than 10 cm^2^. Furthermore, the FTR model allowed for surgical augmentation of the TV annulus using the De-Vega procedure and provided the quantitative assessment of the De-Vega procedure’s influence on hydrodynamic performances and morphology.

Several reports have detailed in vivo, ex vivo, and in vitro tests to evaluate TV function^13,14,31–37^. In vivo animal studies pose significant challenges in producing reproducible FTR conditions. These in vivo models are difficult to manipulate to produce severe FTR, and high costs present another significant issue. In a previous ex vivo study using porcine hearts, flow was generated by a piston pump connected to the explanted hearts via the interventricular septum. Although these models are often used to simulate FTR and test new devices and techniques^38^, they have inherent limitations. Despite the anatomical similarity between porcine and human hearts, the heart muscle in these models cannot pump flow. The use of a piston pump system induces paradoxical motion of the ventricle, resulting in an increase in ventricular volume during systole and a decrease during diastole^13,33^, leading to unphysiological conditions. Moreover, previous ex-vivo studies have struggled to produce a severe grade of FTR model. One exception involved applying two bands to a right heart at the levels of papillary muscles and the TV annulus. However, this method might induce unphysiological behavior in the TV.

In previous in vitro studies, when the TV annulus was installed in test apparatuses in its enlarged state, the annulus was under tension^36^. This tension made it difficult to properly evaluate the performance of new annuloplasty devices or techniques. Such tension in the TV annulus can lead to tearing of the annulus tissue during annuloplasty procedures. Additionally, the production of severe FTR models with a physiologically enlarged TV annulus has not been achieved. Moreover, previous in vitro studies incorporating excised TV tissue into the simulator often overlooked the physiological relative positions between the annulus and papillary muscles^37^.

In this study, we utilized the TV annulus, TV leaflets, chordae tendineae, papillary muscles, and right ventricular wall as one continuous piece of tissue. This approach allowed us to preserve the 3-D anatomical relationships of the “tricuspid complex” in the simulator. We observed a significant enlargement of the distance between the anterior and septal papillary muscles in the FTR model. This finding is consistent with clinical observations, where an enlargement of the distance between the anterior and septal papillary muscles is more frequently reported in patients with FTR^39^. We developed a novel in vitro tissue-silicone integrated right ventricular simulator that incorporates a FTR model with a dilated TV annulus. This was achieved by partially degenerating collagen of the TV annulus and dilating the annulus using a specially designed dilator to mimic the morphology of a human FTR annulus. We demonstrated the feasibility of our original method that induces gradual dilation of the annulus while preventing annular tissue tearing, thus successfully creating a severe FTR model. We believe that this right ventricular circulatory simulator serves as a platform to facilitate the development and pre-clinical study of novel transcatheter TV devices, as well as the evaluation of efficacy and safety of surgical annuloplasty devices and techniques.

This study had some limitations. First, in our right ventricular model, the free wall and septum models deform in the same manner because the driving pressure acts evenly on the outside surface of the right ventricular model. In humans, the RV free wall contracts and dilates predominantly, while the septum does not deform. Second, although we have left a 10-mm tissue margin from the annulus to the right atrial side, the movement of the annulus is limited. Clinically, it has been reported that the TV annulus, which is saddle-shaped during the closing phase, becomes flattened and deformed in patients with severe FTR. However, the FTR model in the present study may not simulate such morphological changes of the TV annulus. Third, we used a 0.9% physiological saline solution as the working fluid. The principle of flow measurement using a cardiology ultrasound system with color Doppler mode tracks red blood cells. Therefore, the accuracy of the jet flow area values might have been compromised. Forth, the effects of the annular augmentation procedure could be evaluated only in the intraoperative phase, where the remodeling effects of the right ventricle were absent. Therefore, the results obtained from the present in vitro model should be carefully translated into clinical practice and should be confirmed with clinical data. A study assessing the correlation between the experimental data using the present model and clinical findings of mid-term follow-ups may contribute to the mid-term prediction of surgical outcomes.

Nevertheless, the FTR models developed in the present study allows for the independent adjustment of parameters affecting treatment outcomes, such as annular diameter, RV volume, RV geometry, and pulmonary artery pressure. In future studies, we will examine the characteristics of FTR with different severity and pathology, particularly with higher right ventricular pressure, larger RV volume, or smaller tethering height of leaflets, which are considered negative indices for surgical outcomes. We believe that the thorough assessment of the effects of tricuspid interventional and surgical treatments in pathological models may contribute to accelerating development and clinical translation of emerging transcatheter and surgical TV devices.

## Conclusions

We have successfully developed a severe and reproducible FTR model using a novel pulsatile circulatory simulator integrated with a tissue-silicone right ventricular model. This innovative model incorporates a porcine dilated TV annulus and ventricular wall as a single continuous piece of tissue. Our approach involved a novel technique for creating a dilated TV annulus by partially degenerating the annulus collagen with an enzymatic solution, followed by its enlargement using a specially designed dilator. This method allowed us to successfully integrate the dilated annulus of RV tissue into a silicone model of dilated right ventricle. The effectiveness of the model was demonstrated by its ability to replicate a severe grade of regurgitant volume, measuring 45 mL/beat, and a flow jet area exceeding 10 cm^2^, indicative of a severe FTR condition. Additionally, the model facilitated the application and quantitative assessment of a surgical annular augmentation procedure on the TV annulus, enabling the evaluation of its impact on hydrodynamic performances and morphological changes. This advancement in creating a severe FTR model may open new avenues for the development and evaluation of transcatheter TV devices and surgical techniques.

### Supplementary Information

Supplementary Information.

## Data Availability

The datasets generated and/or analyzed during the current study are not publicly available due to no public text sources but are available from the corresponding author on reasonable request.
