# Supplementary material for: A tissue-silicone integrated simulator for right ventricular pulsatile circulation with severe functional tricuspid regurgitation
Source: Sci Rep. 2024 Mar 1;14:5120. doi: 10.1038/s41598-024-55058-w (PMC10907752; doi:10.1038/s41598-024-55058-w)
Supplement: Supplementary file 1 — Supplementary Information. [file 41598_2024_55058_MOESM1_ESM.pptx]

## Slide 1
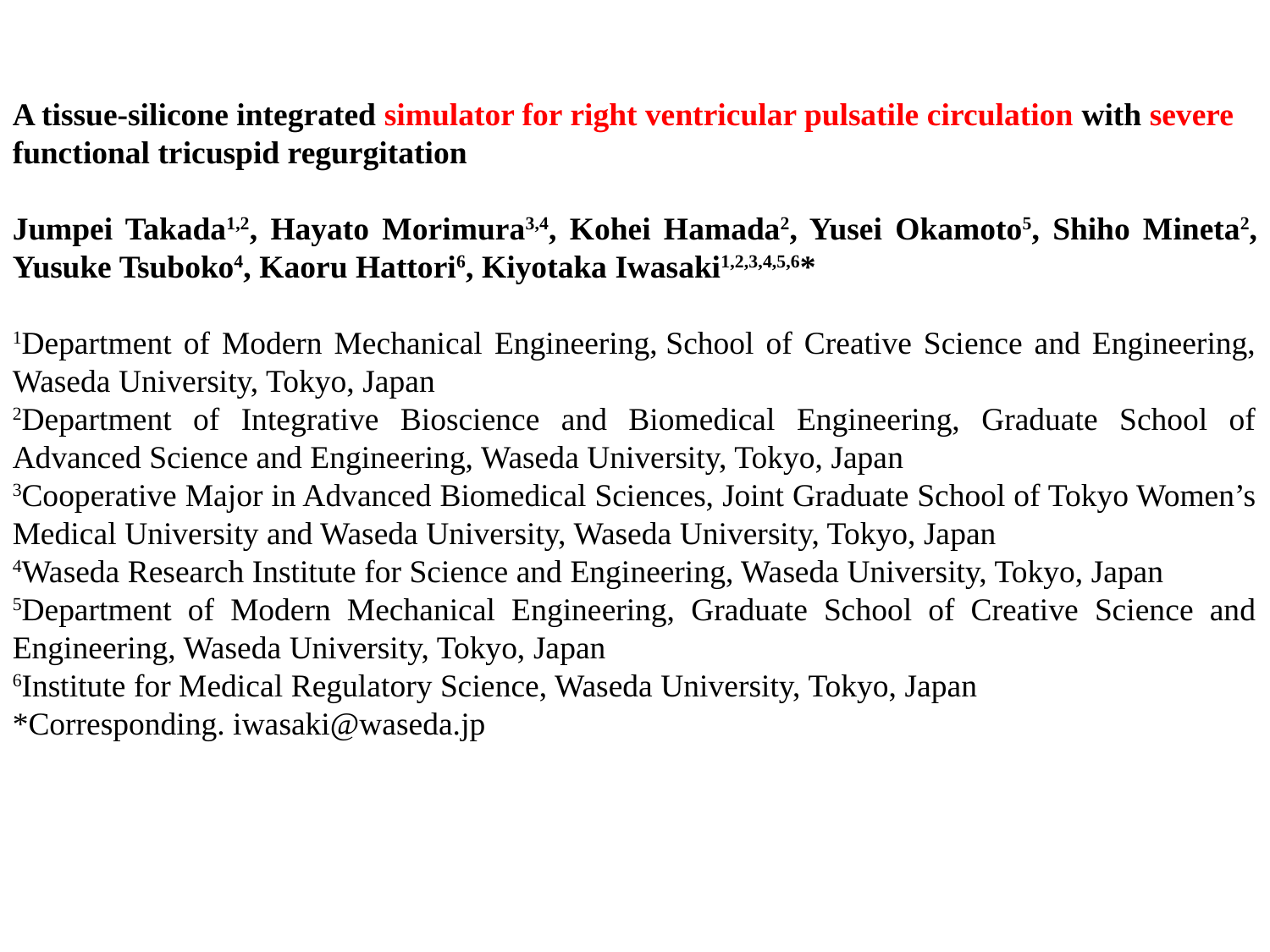

A tissue-silicone integrated simulator for right ventricular pulsatile circulation with severe functional tricuspid regurgitation
Jumpei Takada1,2, Hayato Morimura3,4, Kohei Hamada2, Yusei Okamoto5, Shiho Mineta2, Yusuke Tsuboko4, Kaoru Hattori6, Kiyotaka Iwasaki1,2,3,4,5,6*
1Department of Modern Mechanical Engineering, School of Creative Science and Engineering, Waseda University, Tokyo, Japan
2Department of Integrative Bioscience and Biomedical Engineering, Graduate School of Advanced Science and Engineering, Waseda University, Tokyo, Japan
3Cooperative Major in Advanced Biomedical Sciences, Joint Graduate School of Tokyo Women’s Medical University and Waseda University, Waseda University, Tokyo, Japan
4Waseda Research Institute for Science and Engineering, Waseda University, Tokyo, Japan
5Department of Modern Mechanical Engineering, Graduate School of Creative Science and Engineering, Waseda University, Tokyo, Japan
6Institute for Medical Regulatory Science, Waseda University, Tokyo, Japan
*Corresponding. iwasaki@waseda.jp

## Slide 2
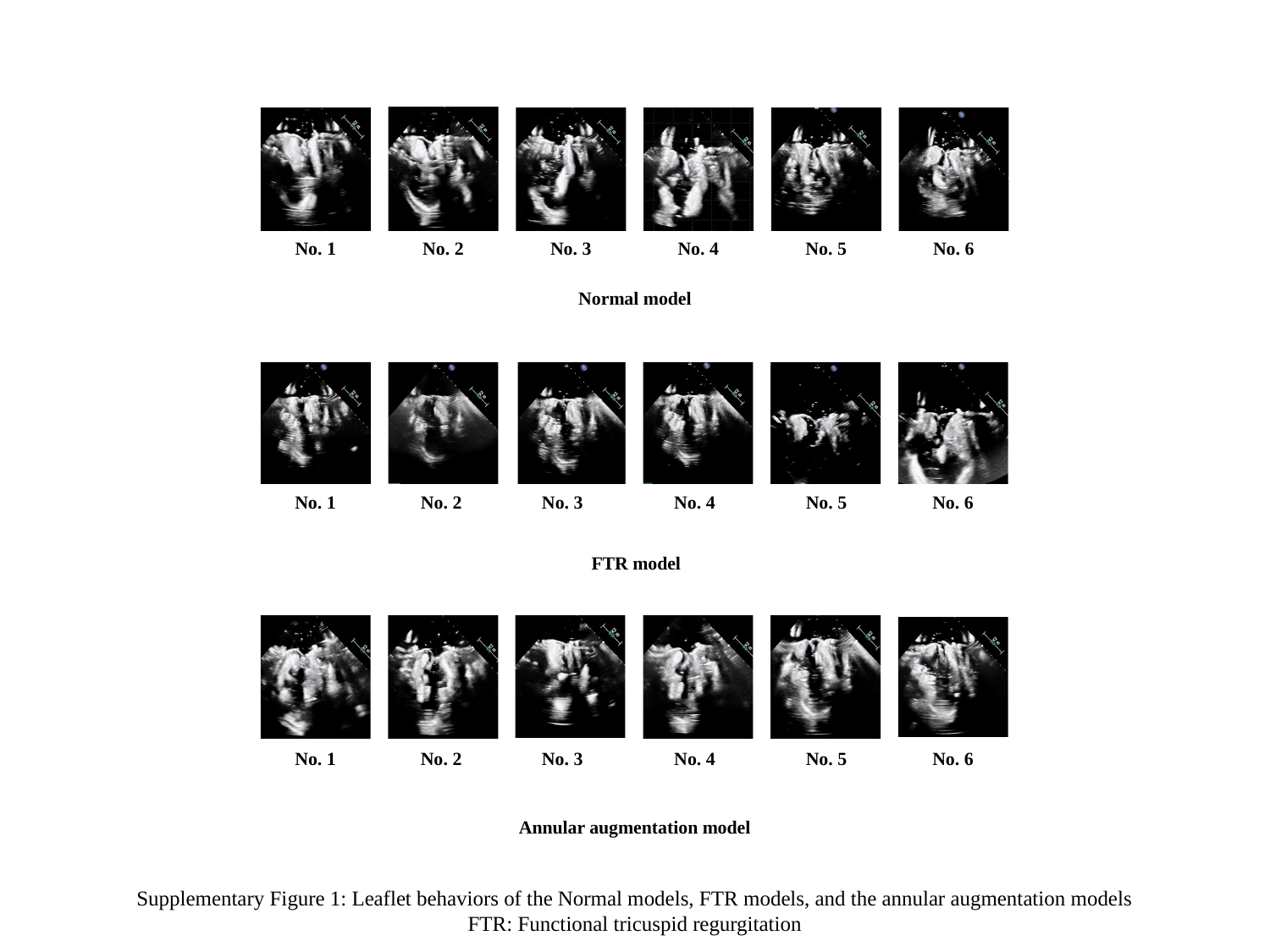

No. 1
No. 2
No. 3
No. 4
No. 5
No. 6
Normal model
No. 1
No. 2
No. 3
No. 4
No. 5
No. 6
FTR model
No. 1
No. 2
No. 3
No. 4
No. 5
No. 6
Annular augmentation model
Supplementary Figure 1: Leaflet behaviors of the Normal models, FTR models, and the annular augmentation models
FTR: Functional tricuspid regurgitation

## Slide 3
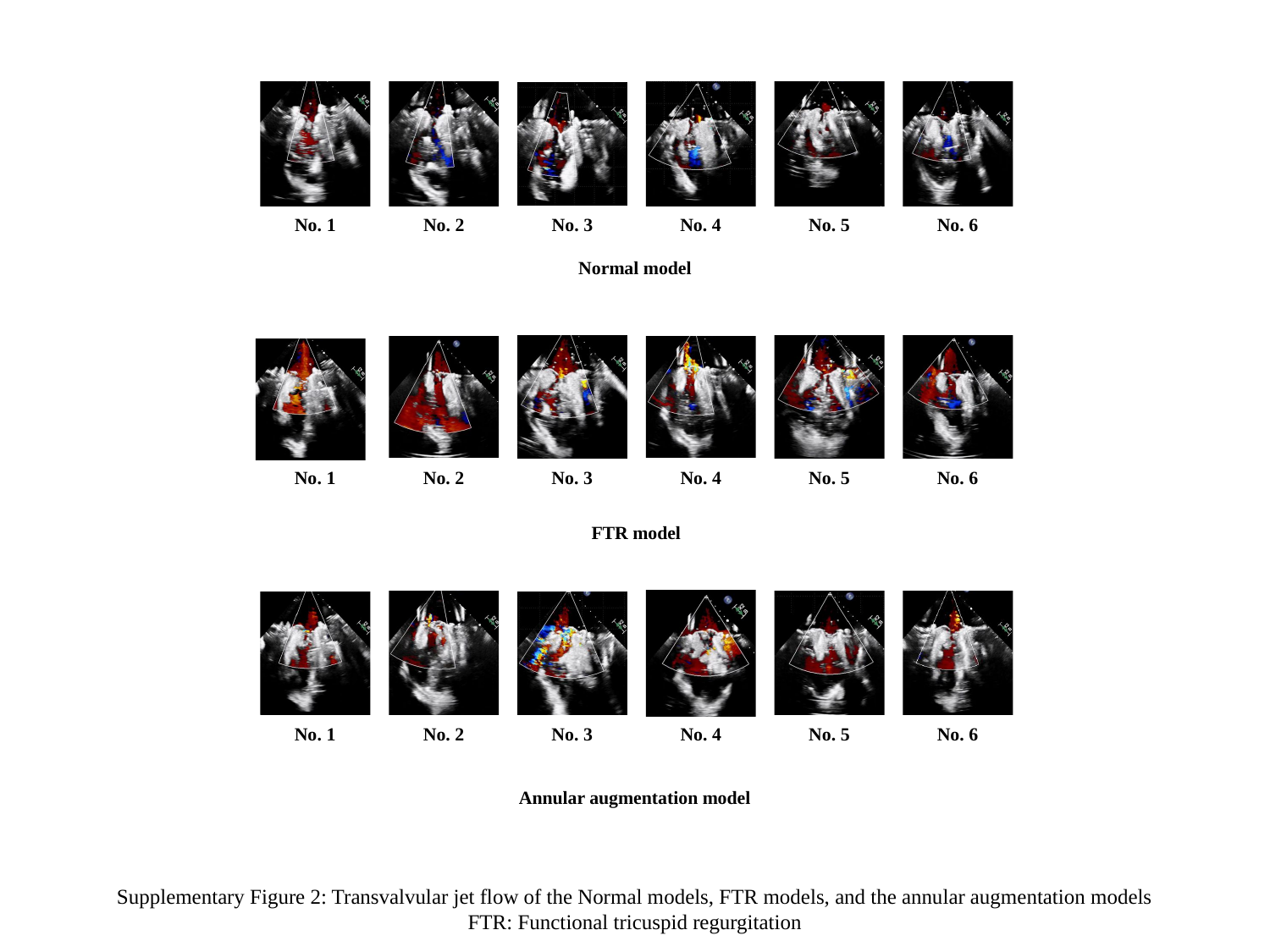

No. 1
No. 2
No. 3
No. 4
No. 5
No. 6
Normal model
No. 1
No. 2
No. 3
No. 4
No. 5
No. 6
FTR model
No. 1
No. 2
No. 3
No. 4
No. 5
No. 6
Annular augmentation model
Supplementary Figure 2: Transvalvular jet flow of the Normal models, FTR models, and the annular augmentation models
FTR: Functional tricuspid regurgitation

## Slide 4
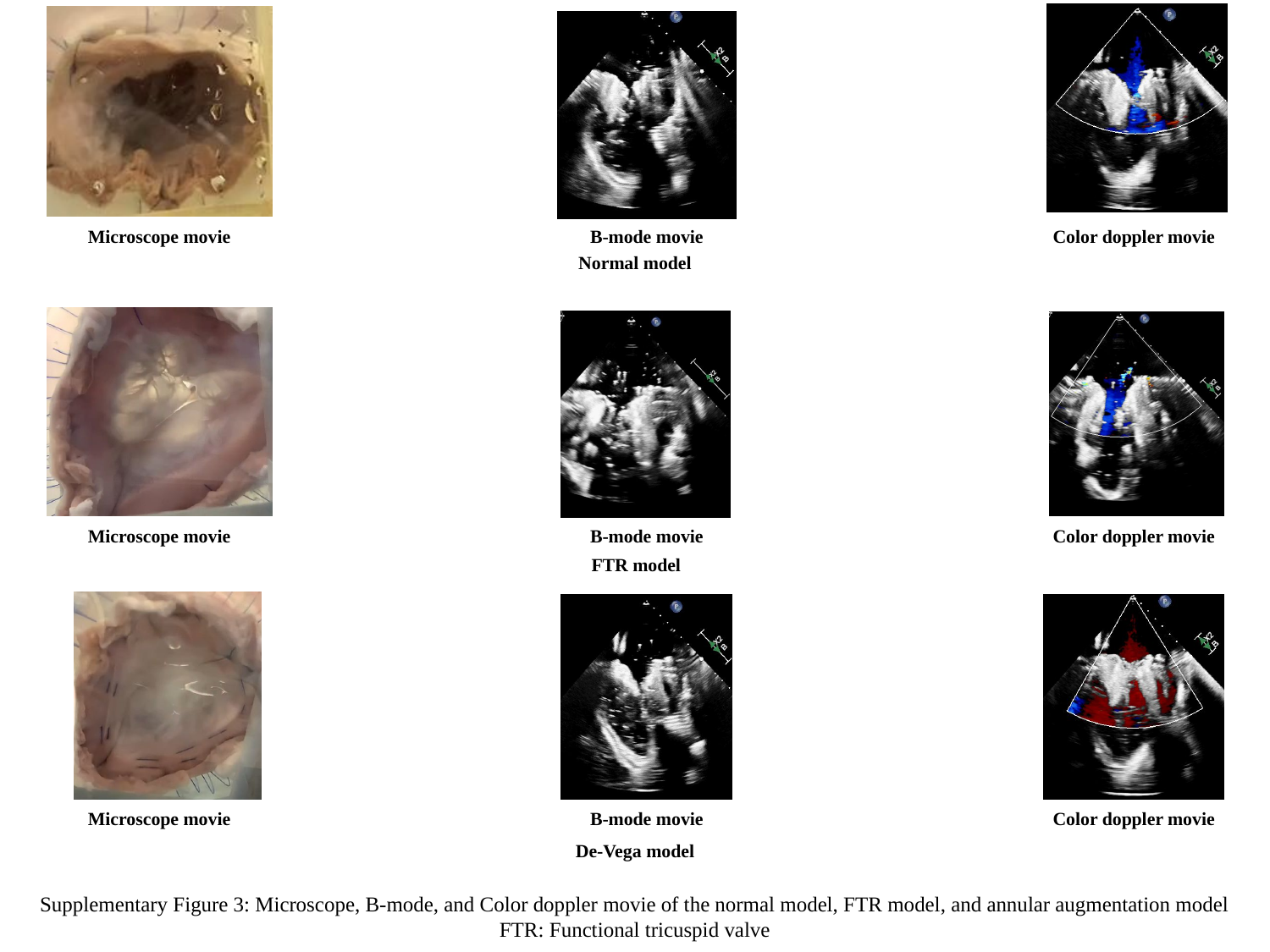

Color doppler movie
B-mode movie
Microscope movie
Normal model
Color doppler movie
B-mode movie
Microscope movie
FTR model
Color doppler movie
B-mode movie
Microscope movie
De-Vega model
Supplementary Figure 3: Microscope, B-mode, and Color doppler movie of the normal model, FTR model, and annular augmentation model
FTR: Functional tricuspid valve
